# The aetiology and incidence of infective endocarditis in people living with rheumatic heart disease in tropical Australia

**DOI:** 10.1007/s10096-023-04641-6

**Published:** 2023-07-20

**Authors:** Andrew Basaglia, Katherine Kang, Rob Wilcox, Alistair Lau, Kylie McKenna, Simon Smith, Ken W. T. Chau, Josh Hanson

**Affiliations:** 1 Department of Medicine, Cairns Hospital, Cairns, QLD Australia; 2 Department of Medicine, Sunshine Coast University Hospital, Birtinya, QLD Australia; 3 Department of Cardiology, Princess Alexandra Hospital, Woolloongabba, QLD Australia; 4 Tropical Public Health Service, Cairns, QLD Australia; 5 James Cook University, Cairns, QLD Australia; 6 Department of Nephrology, Gold Coast University Hospital, Gold Coast, QLD Australia; 7 The Kirby Institute, University of New South Wales, Sydney, Australia

**Keywords:** Infective endocarditis, Rheumatic heart disease, Bacterial sepsis, Skin health, Dental health, Tropical medicine

## Abstract

**Purpose:**

To define the incidence and microbiological aetiology of infective endocarditis (IE) in patients with rheumatic heart disease (RHD) in tropical Australia.

**Methods:**

A retrospective study that examined all episodes of IE between January 1998 and June 2021 among individuals on the RHD register in Far North Queensland, Australia.

**Results:**

There were 1135 individuals with a diagnosis of RHD on the register during the study period, representing 10962 patient-years at risk. Overall, there were 18 episodes of definite IE occurring in 16 individuals, although only 7 episodes occurred in native valves (11 occurred in prosthetic valves) equating to 0.7 episodes of native valve IE/1000 patient-years. No patient with mild RHD - and only one child with RHD - developed IE during the study period. Despite the study’s tropical location, the causative organism was usually typical skin or oral flora. Among individuals with an indication for benzathine penicillin G (BPG) prophylaxis, only 1/6 episodes of IE due to a penicillin-susceptible organism received BPG in the month before presentation.

**Conclusion:**

Although RHD predisposes individuals to IE, the absolute risk of IE in native valve disease in tropical Australia is low and might be reduced further by improved adherence to secondary BPG prophylaxis.

**Supplementary Information:**

The online version contains supplementary material available at 10.1007/s10096-023-04641-6.

## Introduction

The case-fatality rate of infective endocarditis (IE)—infection of the endocardial surface of the heart—can approach 30% [1]. Even those who survive the infection can face devastating complications that include stroke, heart failure, or a requirement for valve replacement surgery [2]. A number of factors predispose individuals to developing IE; the most important of these is underlying structural heart disease [3]. Globally, this is most commonly rheumatic heart disease (RHD), although in high-income countries the proportion of RHD-related IE cases has declined significantly recently as the prevalence of RHD has fallen in these countries [4].

Despite its status as a high-income country with a universal health system, Australia continues to have a significant burden of RHD with approximately 80% of cases occurring in its Aboriginal and Torres Strait Islander people (hereafter respectively referred to as Indigenous Australians) [5]. Despite targeted efforts, in Far North Queensland (FNQ) in northern Australia, the annual incidence of RHD is increasing and is strongly associated with the socioeconomic disadvantage experienced by many Indigenous Australians in the region [6, 7]. The socioeconomic disadvantage experienced by Indigenous Australians in the region also leads to a disproportionate burden of many infectious diseases, evolving antimicrobial resistance and poorer skin and dental health, all factors that might be expected to impact on the local incidence and aetiology of IE [8–11].

The primary aim of secondary prophylaxis with benzathine penicillin G (BPG) is to prevent the recurrence of Group A Streptococcal infections and the subsequent development or progression of valvular disease [12]. However, there may potentially be added protection against serious infection—including IE—due to penicillin-sensitive organisms. This study was performed to determine the local incidence and characteristics of IE among people living with RHD in the FNQ region of tropical Australia where the prevalence of RHD in some communities is greater than 2.5% [6]. The study aimed to identify the microbiological aetiology of confirmed IE cases; it also evaluated episodes of bacteremia in patients with RHD without IE to provide a crude estimate of the likelihood of IE complicating an episode of bacteremia. Finally, the study examined the impact of prophylactic BPG for secondary prevention of ARF/RHD in reducing the burden of IE and other serious infections in the region’s RHD patients. It was hoped that these data would define an RHD patient’s absolute risk for developing IE and might be used to inform the optimal clinical management of these patients.

## Methods

This retrospective study was performed at Cairns Hospital, the sole tertiary referral hospital that serves FNQ, a region of 380,000 km^2^ in Northeast tropical Australia. The region is home to approximately 290,000 people, 17% of whom identify as Indigenous Australians.

All individuals with ARF and RHD diagnoses are recorded in the Queensland ARF/RHD register; ARF and RHD have been notifiable diseases in the state of Queensland since 1999 and 2018, respectively. Investigators used the Queensland ARF/RHD register to identify patients who were eligible for study inclusion if they had a diagnosis of RHD confirmed on an echocardiogram that had been reported by a specialist physician between 1 January 1998 and 30 June 2021; the register defines RHD severity using these reports. If an individual has had a history of RHD that required a prosthetic heart valve, they are automatically defined as having severe RHD. The patients’ demographics, echocardiogram findings, and details of any cardiac surgery were also collected from the Queensland ARF/RHD register which was used to determine the number of years that an individual with confirmed RHD was “at risk” of IE. Individuals living in the Torres and Cape Hospital and Health Service—an area that includes the Cape York Peninsula and the Torres Strait Islands—were said to have a remote residence.

Secondary prophylaxis data were also collected from the RHD register, although complete secondary prophylaxis data were only available from 2012 and so adherence to secondary prophylaxis could only be assessed reliably from this date. Adherence to secondary prophylaxis was determined by dividing the number of doses of BPG received by the number prescribed and expressed as a percentage.

The state’s health information system—HIBISCUS—was used to identify all hospitalizations between 1 January 1998 and 30 June 2021, which were classified using International Statistical Classification of Diseases and Related Health Problems (ICD-10) coding. Episodes of bacteremia that occurred during these hospitalizations were identified using the State’s electronic laboratory database, AUSLAB. Infective endocarditis was defined using modified Duke criteria; only cases where the diagnosis was “definite” were included as IE cases [2]. Patient medical records and AUSLAB were examined to determine if these criteria were satisfied; if a criterion was not documented, it was presumed to be absent. The medical record of individuals with IE was also examined to assess BPG adherence.

De-identified data were entered into an electronic database (Microsoft Excel 2016, Microsoft, Redmond, WA, USA) and analyzed using statistical software (Stata version 14.2, StataCorp LLC, College Station, TX, USA). Groups were compared using Fisher’s exact test and the chi-squared test, where appropriate.

## Results

There were 1135 individuals on the RHD register with a diagnosis of RHD at some point in the study period, representing 10,962 patient-years at risk. Overall, there were 18 hospitalizations in 16 of these individuals that satisfied modified Duke criteria for definite IE (2 individuals had 2 distinct episodes of IE), representing 1.6 episodes of IE per 1000 patient-years. There were 7 episodes of native valve IE in 9810 patient-years of risk: equating to a risk of 0.7 episodes of native valve IE per 1000 patient-years. There were 11 episodes of prosthetic valve IE in 1152 patient-years of risk: equating to 9.5 episodes per 1000 patient-years.

Most episodes of IE were caused by skin or oral flora, with *S. aureus* the most common (Table 1). There was only 1 episode of a gram-negative IE (1 case of *Haemophilus parainfluenzae*) and no cases of fungal IE during the study period. All 16 individuals survived their episode(s) of IE, although 3 patients died subsequently: 2 directly from RHD-related complications and 1 from an unrelated illness.

**Table 1:** Blood culture isolates in the cohort stratified by the presence or absence of confirmed infective endocarditis or a prosthetic heart valve

Isolates in cases of definite native valve IE (*n* = 7)	Isolates in cases of definite prosthetic valve IE (*n* = 11)	Isolates in cases of bacteremia without definite IE or a prosthetic valve (*n* = 75)	Isolates in cases of bacteremia without definite IE in individuals with a prosthetic valve (*n* = 26)
*Staphylococcus aureus* (*n* = 3) ^a^ Group C Streptococcus (*n* = 1) *Haemophilus parainfluenzae* (*n* = 1) *Streptococcus mitis* (*n* = 1) *Corynebacterium diphtheriae* (*n* = 1) ^b^	*Staphylococcus aureus* (*n* = 4) ^c^ *Staphylococcus epidermidis* (*n* = 1) ^d^ *Streptococcus pyogenes* (*n* = 1) *Aggregatibacter aphrophilus* (*n* = 1) *Streptococcus mitis* (*n* = 1) *Streptococcus oralis* (*n* = 1) *Streptococcus constellatus* (*n* = 1) *Enterococcus faecalis* (*n* = 1) ^c^	*Escherichia coli* (*n* = 18) *Staphylococcus aureus* (*n* = 11) ^e^ *Streptococcus pyogenes* (*n* = 7) *Klebsiella pneumoniae* (*n* = 5) *Streptococcus pneumoniae* (*n* = 5) *Acinetobacter baumanii* (*n* = 4) *Burkholderia pseudomallei* (*n* = 2) *Haemophilus influenzae* (*n* = 2) *Streptococcus agalactiae* (*n* = 2) *Streptococcus mitis* (*n* = 2) *Achromobacter xylosoxidans* (*n* = 1) *Acinetobacter lwoffii* (*n* = 1) *Burkholderia cepacia* (*n* = 1) *Citrobacter freundii* (*n* = 1) *Enterobacter aerogenes* (*n* = 1) *Klebsiella oxytoca* (*n* = 1) *Listeria monocytogenes* (*n* = 1) *Moraxella species* (*n* = 1) *Proteus penneri* (*n* = 1) *Serratia marcescens* (*n* = 1) *Staphylococcus capitis* (*n* = 1) *Staphylococcus lugdunensis* (*n* = 1) *Streptococcus anginosus* (*n* = 1) *Streptococcus constellatus* (*n* = 1) *Streptococcus dysgalactiae* (*n* = 1) *Streptococcus parasanguinis* (*n* = 1) *Streptococcus salivarius* (*n* = 1)	*Escherichia coli* (*n* = 5) *Staphylococcus aureus* (*n* = 4) ^f^ *Streptococcus pneumoniae* (*n* = 3) *Streptococcus mitis* (*n* = 2) *Acinetobacter baumanii* (*n* = 1) *Aggregatibacter actinomycetemcomitans* (*n* = 1) *Enterobacter aerogenes* (*n* = 1) *Enterobacter cloacae* (*n* = 1) *Enterococcus faecalis* (*n* = 1) *Klebsiella pneumoniae* (*n* = 1) *Morganella morganii* (*n* = 1) *Stenotrophomonas maltophilia* (*n* = 1) *Streptococcus agalactiae* (*n* = 1) *Streptococcus gordonii* (*n* = 1) *Streptococcus pyogenes* (*n* = 1) *Streptococcus sanguinis* (*n* = 1)

^a ^2 were methicillin-resistant *S. aureus*

^b^ Non-toxigenic *C. diphtheriae*

^c^ One individual had two episodes of methicillin-sensitive *S. aureus* prosthetic valve endocarditis 5 years apart

^d^ One individual had two episodes of prosthetic valve endocarditis 5 months apart with *E. faecalis* and *S. epidermidis*, respectively

^e^ 6 were methicillin-resistant *S. aureus*

^f ^1 was methicillin-resistant *S. aureus*

There were 97 episodes of bacteremia among individuals with RHD that did not satisfy the criteria for a definite IE diagnosis; in 4 of these episodes, two organisms were isolated. Of the 97 episodes, 72 (74%) occurred in patients with native valves, equating to a risk of 7.3 episodes per 1000 patient-years. Meanwhile, 25 (26%) occurred in patients with prosthetic valves, equating to 21.7 episodes per 1000 patient-years. *Escherichia coli* was the most commonly isolated pathogen, but skin and oral flora were also seen frequently (Table 1). Overall, 7/22 (32%) cases of *S. aureus* bacteremia represented definite IE, and 4/7 (57%) of these episodes of IE occurred in patients with prosthetic valves. Meanwhile, 4/14 (29%) episodes of viridans group streptococcal bacteremia represented definite IE, and 3/4 (75%) of these episodes of IE occurred in prosthetic valves.

When compared with the characteristics of the 832 FNQ residents with confirmed RHD on the RHD register at the end of the study period, who had no history of IE, only RHD severity was statistically associated with the development of IE (Table 2). The association with severe RHD was largely explained by the presence of a prosthetic heart valve in 11/12 episodes of definite IE that occurred in patients with severe RHD. It was notable that no patient with mild RHD was diagnosed with IE during the study period. Only one child with RHD developed IE during the study period.

**Table 2:** Comparison of the individuals living with RHD who developed infective endocarditis and those that did not

	No infective endocarditis *n* = 832 ^a^	Infective endocarditis *n* = 16 ^b^	*p*
Median (IQR) age (years)	45 (30–61)	38 (29–56)	0.31
Female sex ^c^	586 (70%)	9 (56%)	0.27
Indigenous Australian	687 (83%)	14 (88%)	1.0
Remote residence	358 (43%)	4 (25%)	0.13
Mild RHD	266 (32%)	0	0.004
Moderate RHD	277 (33%)	6 (38%)	0.79
Severe RHD	289 (35%)	10 (63%)	0.03
Any prosthetic valve	166 (21%)	9 (56%)	0.002
Mechanical valve	120 (14%)	3 (19%)	0.72

*RHD*, rheumatic heart disease; *IQR*, interquartile range

^a^ Individuals with confirmed RHD on the RHD register at the end of the study period with no history of IE

^b^ Eighteen episodes of IE occurred in 16 patients during the study period; characteristics at first episode presented

^c^ Biological; defined at birth

Patients with bacteremia without IE were more likely to have mild RHD than the patients with IE (24/74 (32%) versus 0/16, *p* = 0.005) and less likely to have a prosthetic valve (20/74 (27%) versus 9/16 (56%), *p* = 0.04) (supplementary table 1).

### BPG adherence and impact on infection

Australia’s 2020 guidelines would have recommended that in 10/18 (56%) episodes of IE, the affected individuals should have been receiving BPG for secondary ARF/RHD prophylaxis [12]. The isolate in 6/10 of these IE cases was penicillin susceptible. However, in only 1/6 of these episodes was BPG administered in the month prior to the IE diagnosis (Table 3).

**Table 3:** Adherence to BPG recommendations in patients with infective endocarditis, stratified by the isolate’s penicillin susceptibility

Isolate	Penicillin sensitivity ^a^	BPG recommended ^b^	BPG administered in the 1 month prior to admission
*Streptococcus mitis*	Sensitive	Yes	Yes
*Enterococcus faecalis*	Sensitive	Yes	No
*Haemophilus parainfluenzae*	Sensitive	Yes	No
*Streptococcus pyogenes*	Sensitive	Yes	No
*Streptococcus oralis*	Sensitive	Yes	No
*Corynebacterium diphtheriae*	Sensitive	Yes	No
*Staphylococcus aureus*	Sensitive	No	No
*Streptococcus mitis*	Sensitive	No	No
Group C Streptococcus* sp*	Sensitive	No	No
*Streptococcus constellatus*	Sensitive	No	No
*Aggregatibacter aphrophilus*	Sensitive	No	No
*Staphylococcus epidermidis*	Resistant	Yes	No
*Staphylococcus aureus* (MRSA)	Resistant	Yes	No
*Staphylococcus aureus* (MRSA)	Resistant	Yes	No
*Staphylococcus aureus*	Resistant	Yes	Yes
*Staphylococcus aureus*	Resistant	No	No
*Staphylococcus aureus*	Resistant	No	Yes
*Staphylococcus aureus*	Resistant	No	No

*BPG*, benzyl penicillin G; *MRSA*, methicillin-resistant *Staphylococcus aureus*

^a^ Defined by Vitek ® 2 (bioMérieux, France) or *E*-test using EUCAST guidelines where appropriate

^b^ In current Australian RHD guidelines[12]

Australia’s 2020 guidelines would have recommended that in 29/97 (30%) episodes of non-IE bacteremia, the affected individuals should have been receiving BPG for secondary ARF/RHD prophylaxis [12]. In these 29 episodes, there were 32 bacterial isolates, 15 were penicillin susceptible, in 11 of these patients bicillin data were available; only one of these 11 patients was BPG administered in the month prior to the IE diagnosis (Table 4). At the end of the study period, there were 768 individuals in the region receiving BPG prophylaxis, 350 (46%) with confirmed RHD. The median (IQR) BPG adherence in individuals receiving BPG prophylaxis was 54% (31–77); the median (IQR) BPG adherence among individuals in the cohort with confirmed RHD was also 54% (31–77).

**Table 4:** Recommendation for—and adherence to—BPG in individual episodes of bacteremia without infective endocarditis with a penicillin-sensitive isolate

Isolate ^a^	BPG recommended ^b^	BPG administered 1 month prior to admission
*Moraxella sp*	Yes	No
*Staphylococcus capitis*	Yes	Unknown ^c^
*Streptococcus agalactiae*	Yes	No
*Streptococcus constellatus*	Yes	Yes
*Streptococcus gordonii*	Yes	No
*Streptococcus mitis*	Yes	No
*Streptococcus mitis*	Yes	No
*Streptococcus pneumoniae*	Yes	Unknown ^c^
*Streptococcus pneumoniae*	Yes	No
*Streptococcus pyogenes*	Yes	No
*Streptococcus pyogenes*	Yes	No
*Streptococcus pyogenes*	Yes	Unknown ^c^
*Streptococcus salivarius*	Yes	Unknown ^c^
*Streptococcus sanguinis*	Yes	No
*Streptococcus anginosus*	Yes	No
*Aggregatibacter actinomycetemcomitans*	No	No
*Enterococcus faecalis*	No	Unknown ^c^
*Haemophilus influenzae* (β-lactamase neg)	No	No
*Listeria monocytogenes*	No	Unknown ^c^
*Streptococcus agalactiae*	No	No
*Streptococcus agalactiae*	No	No
*Streptococcus dysgalactiae*	No	Unknown ^c^
*Streptococcus mitis*	No	No
*Streptococcus mitis*	No	Unknown ^c^
*Streptococcus parasanguinis*	No	No
*Streptococcus pneumoniae*	No	No
*Streptococcus pneumoniae*	No	No
*Streptococcus pneumoniae*	No	No
*Streptococcus pneumoniae*	No	No
*Streptococcus pneumoniae*	No	Unknown ^c^
*Streptococcus pneumoniae*	No	Unknown ^c^
*Streptococcus pyogenes*	No	Unknown ^c^
*Streptococcus pyogenes*	No	No
*Streptococcus pyogenes*	No	No
*Streptococcus pyogenes*	No	No
*Streptococcus pyogenes*	No	No

*BPG* benzathine penicillin G

^a^ The 36 penicillin-sensitive organisms are listed alphabetically, but are stratified by whether they were identified in patients in whom BPG was recommended in current Australian guidelines; isolates occurring in individuals meeting criteria for BPG prophylaxis are listed first. There were 65 isolates in cases of bacteremia that were not penicillin-sensitive: *Achromobacter xylosoxidans* (1), *Acinetobacter baumanii* (5), *Acinetobacter lwoffii* (1), *Burkholderia cepacia* (1), *Burkholderia pseudomallei* (2), *Citrobacter freundii* (1), *Enterobacter aerogenes* (2), *Enterobacter cloacae* (1), *Escherichia coli* (23), *Haemophilus influenzae (β-lactamase positive)* (1), *Klebsiella pneumoniae* (6), *Klebsiella oxytoca* (1), *Morganella morganii* (1), *Proteus penneri* (1), *Serratia marcescens* (1), *Staphylococcus aureus* (8), *Staphylococcus aureus (MRSA)* (7), *Staphylococcus lugdunensis* (1), *Stenotrophomonas maltophilia* (1)

^b^ In current Australian RHD guidelines[12]

^c^ Episode of bacteremia occurred prior to 2012; reliable BPG adherence data not available

### Other hospitalizations

The 1135 individuals with a diagnosis of RHD on the register had 9050 acute hospitalizations during the study period equating to 825 per 1000 patient-years. Of these 9050 hospitalizations, 1148 were cardiac-related, and there were 120 admissions with a stroke or transient ischaemic attack. Admissions with infection were common: there were 1273 admission for non-IE infections, including 470 respiratory tract infections and 112 urinary tract infections (supplementary table 2).

## Discussion

RHD-related IE is a rare diagnosis in FNQ despite the significant and increasing burden of RHD in the region, a high local incidence of skin sepsis, and the well-documented challenges with delivering optimal dental care to the communities where RHD prevalence is highest [6, 8, 13]. The risk of native valve IE in FNQ patients with RHD was only 0.7/1000 patient-years of risk, and it was notable that in the 23 years of the study, there were no cases of IE in patients with mild RHD. Instead, almost two-thirds of IE cases occurred in patients with prosthetic heart valves. Furthermore, despite the grave prognosis of IE—and of prosthetic valve IE, in particular—it was heartening that there were no deaths from IE in the cohort during the study period.

The incidence of RHD-related IE is lower in our cohort than the overall rate of 4.3/1000 patient-years reported in a data-linkage series from the Northern Territory, a region with one of the highest reported RHD rates in the world and where the RHD prevalence in some communities approaches 10% [5, 14]. The IE cases in this NT series were defined using ICD hospitalization coding, rather than strict use of the Duke criteria and did not distinguish between native or prosthetic valve IE. However, while both series demonstrate that RHD is a significant risk factor for IE—the annual incidence of IE in temperate Australia is, by comparison, estimated to be 4.7/100,000 [15]—they suggest that IE remains an uncommon complication of RHD in tropical Australia. This observation is supported by another study that examined infective endocarditis in northern Australia [16]. This study examined IE cases in FNQ (over a period of 5.8 years) and The Top End of the Northern Territory of Australia (over a 4-year period). Although the study demonstrated that RHD was a risk factor for IE, only 8/89 (9%) cases of IE in the two jurisdictions during the study period occurred in patients with native valve RHD [16]. In the current study, the rate of bacteremia in patients with native valve RHD was 7.3 episodes per 1000 patient-years compared to the rate of definite IE of 0.7 per 1000 patient-years.

A variety of bacteria can cause IE, but *Staphylococcus aureus* (31%), viridans group streptococci (17%), and enterococci (11%) are the organisms most commonly implicated [17]. Despite the tropical setting of our study and the remote, rural residence of many of the patients living with RHD, the isolates in this cohort and prior cohorts [16] were very similar to those from international series [17]. Most of the organisms causing IE in RHD patients represent skin flora—most frequently *S. aureus*—with oral streptococci also common.

The finding of a high incidence of skin and oral flora causing bacteremia in the cohort emphasizes the ongoing challenges with optimizing skin and dental care in the region [18, 19]. Suboptimal skin health is one of the key drivers for RHD in the region and other complications including sepsis and post-streptococcal glomerulonephritis [13, 20–22]. And while there has been much discussion about the role of antibiotic prophylaxis for dental procedures, most cases of IE from oral streptococci are unrelated to procedures and are instead linked to the significant burden of oral disease that is unaddressed [23, 24]. Unfortunately, many people living with RHD struggle to access the oral health and prevention programs that they require, with a lack of confidence in oral health services and the financial burden of practicing optimal preventative care among the explanations [18]. Although there is undoubtedly a need for far greater investment in dental services in our region, it is important to note that dentists in FNQ routinely follow national guidelines in providing antibiotic prophylaxis prior to dental procedures and that this is likely to reduce the IE burden [24].

It was hypothesized that regular administration of BPG for secondary prophylaxis of ARF/RHD might also reduce the incidence of IE from penicillin-susceptible bacteria, and despite the generally poor adherence to BPG in the cohort, there are data in this study to support this theory. There was only a single episode of IE with a penicillin-susceptible isolate and a single case of bacteremia with a penicillin-susceptible isolate in patients who received BPG in the prior month. The fact that secondary prophylaxis slows progression to more advanced valvular disease—and in this cohort, only individuals with moderate or severe RHD developed IE—means that secondary prophylaxis of ARF/RHD has the potential to reduce IE incidence via two separate mechanisms [25]. Of course, this would only have a direct effect on the incidence of IE due to penicillin-sensitive organisms, and, with current dosing regimens, monthly BPG would be unlikely to achieve minimum inhibitory concentrations for all penicillin-sensitive organisms during the entire injection interval [26].

However, despite the established and potential benefits of BPG secondary prophylaxis, the adherence of many individuals in the cohort was suboptimal. Even at the end of the study period after the expansion of the local RHD service [6], the median adherence was only 54%. Exploration of the reasons for this non-adherence was beyond the scope of the study, although previous research has identified that being older, having less severe disease, increasing time since first diagnosis, experience of assault, and hazardous alcohol use are predictors of lower adherence [27]. Secondary prophylaxis of RHD reduces disease progression and may be associated with reduced overall mortality [25]; the likelihood that it reduces the risk of significant infections—including IE—may be a further reason to encourage treatment uptake.

Although the incidence of IE was relatively low in the cohort, it was notable that individuals with RHD were hospitalized far more frequently—over 500 times more frequently—for other reasons. Although most individuals in the cohort were younger than 50, comorbidity was common. This highlights the importance of integrating the management of RHD and these comorbidities—and avoidance of disease siloing—although the challenges in providing longitudinal holistic care to populations with RHD and complex comorbidity are recognized [28]. Of course, the burden of RHD and comorbidity in the cohort are almost certainly explained by the social determinants of health and the ongoing disadvantage that many Indigenous Australia in the region continue to experience [6, 11]. Remedying this situation is an ongoing challenge, but solutions with a biomedical focus are unlikely to have a major impact on health outcomes. Progress is only likely to be achieved through developing community-led and co-designed programs that acknowledge the social and environmental determinants of health that create the necessary milieu for RHD and other diseases of disadvantage [12, 29].

This retrospective study has several important limitations. It almost certainly underestimates the burden of RHD in the FNQ region, although determination of the true RHD prevalence would require comprehensive community screening which has not been logistically feasible across this 380,000km^2^ region [7]. Patients were identified using the Queensland RHD registry, which is likely to have missed some patients as RHD was only declared a notifiable disease in 2018. However, our findings are reported as episodes per 1000 patient-years of follow up which controls for this to some degree and the findings are similar to a prior study in the region that found native valve RHD in less than 10% of IE patients [16]. There are inherent limitations in using ICD coding to define the underlying cause of a patient’s hospitalization, and although this was controlled for by checking blood cultures results from all hospitalizations, this may still underestimate the incidence of culture-negative IE [30]. It is also possible that we have underestimated the incidence of IE in the cohort by only including cases of definite IE. BPG administration records prior to 2012 were incomplete, limiting the reliability of these data, although access to almost a decade of registry data after this time is likely to have mitigated the impact of this issue on our findings. The current 2020 guidelines for RHD management were used to define the requirement for BPG prescription, and these evolved over the study period as the evidence base for optimal RHD management expanded, although this is again unlikely to have had a significant impact on our findings. The study’s findings are not necessarily generalizable to other geographical locations—particularly outside Australia—however, a focus on the social determinants of health and implementation of strategies to improve adherence to secondary prophylaxis would also be expected to be beneficial in these locations.

## Conclusions

Despite the significant RHD burden, high rates of skin and soft tissue infection, and challenges in the delivery of optimal dental care, RHD-related IE remains an uncommon diagnosis in this part of Australia. Improvements in BPG adherence would be expected to both slow the progression of valve disease and reduce the incidence of sepsis—including IE—from penicillin-sensitive organisms. However, a significant improvement in health outcomes is unlikely without addressing the underlying social determinants of health that drive the incidence of these infections and the many non-communicable diseases that remain so prevalent in Aboriginal and Torres Strait Islander communities in the region.

## Supplementary Information

Below is the link to the electronic supplementary material.

Supplementary file1 (DOCX 25 KB)

Supplementary file2 (DOCX 31 KB)

## Data Availability

Data cannot be shared publicly because of the Queensland Public Health Act 2005. Data are available from the Far North Queensland Human Research Ethics Committee (contact via email at FNQ_HREC@health.qld.gov.au) for researchers who meet the criteria for access to confidential data.
