# Supplementary material for: The aetiology and incidence of infective endocarditis in people living with rheumatic heart disease in tropical Australia
Source: Eur J Clin Microbiol Infect Dis. 2023 Jul 20;42(9):1115–23. doi: 10.1007/s10096-023-04641-6 (PMC10427705; doi:10.1007/s10096-023-04641-6)
Supplement: Supplementary file 1 — Supplementary file1 (DOCX 25 KB) [file 10096_2023_4641_MOESM1_ESM.docx]

**Supplementary table 1.** Comparison of the individuals living with RHD who developed definite infective endocarditis and those that had bacteraemia without IE.

|  | Infective endocarditis n=16 ^a^ | Bacteraemia n=74 ^b^ | p |
| --- | --- | --- | --- |
| Median (IQR) age (years) | 38 (29-56) | 49 (37-62) | 0.11 |
| Female sex ^c^ | 9 (56%) | 51 (69%) | 0.39 |
| Indigenous Australian | 14 (88%) | 60 (81%) | 0.73 |
| Remote residence | 4 (25%) | 20 (27%) | 1.0 |
| Mild RHD | 0 | 24 (32%) | 0.005 |
| Moderate RHD | 6 (38%) | 21 (28%) | 0.55 |
| Severe RHD | 10 (63%) | 29 (39%) | 0.10 |
| Any prosthetic valve | 9 (56%) | 20 (27%) | 0.04 |
| Mechanical valve | 3 (19%) | 15 (20%) | 1.0 |

RHD: Rheumatic heart disease; IQR: Interquartile range.

^a^ 18 episodes of IE occurred in 16 patients during the study period; characteristics at first episode presented.

^b^ 97 episodes in 74 patients. Characteristics at first presentation presented.

^c^ Biological; defined at birth.
