# Supplementary material for: The aetiology and incidence of infective endocarditis in people living with rheumatic heart disease in tropical Australia
Source: Eur J Clin Microbiol Infect Dis. 2023 Jul 20;42(9):1115–23. doi: 10.1007/s10096-023-04641-6 (PMC10427705; doi:10.1007/s10096-023-04641-6)
Supplement: Supplementary file 2 — Supplementary file2 (DOCX 31 KB) [file 10096_2023_4641_MOESM2_ESM.docx]

**Supplementary table 2.** Other hospitalisations during the study period

Individuals with confirmed RHD on the Queensland RHD register had a total of 14996 hospitalisations in the local health service between 1^st^ January 1998 – 30^th^ June 2021. After exclusion of 5746 routine admissions for haemodialysis, there were 9050 individual acute hospital admissions, translating to a mean of 8.0 hospitalisations per individual with RHD during the study period. Hospitalisation episodes were categorised by specialty and the most common diagnoses - defined using International Classification of Disease (ICD) - 10 are presented below:

| Specialty/subcategories | Total hospitalisations (n=9050) |
| --- | --- |
| Cardiovascular  Congestive Heart Failure  Atrial Fibrillation/Flutter  Acute Myocardial Infarction  Other cardiovascular diagnoses | 1028  284  145  93  506 |
| Neurology  Seizure/Convulsions  Stroke/Transient Ischaemic Attack  Migraine  Other neurological diagnoses | 301  141  120  12  28 |
| Renal  End-stage Renal Failure  Acute Kidney Injury  Tubulointerstitial Nephritis  Other renal diagnoses | 200  59  38  37  66 |
| Respiratory  Exacerbation of chronic obstructive pulmonary disease  Asthma  Aspiration Pneumonitis  Other respiratory diagnoses | 172  50  35  15  72 |
| Otolaryngology  Otitis Media  Perforation of Tympanic Membrane  Cholesteatoma of middle ear  Other otolaryngology diagnoses | 69  18  14  5  32 |
| Infectious Diseases  Pneumonia  Respiratory tract infections  Cellulitis  Urinary Tract Infection  Other infectious diseases diagnosis | 1289  260  210  117  112  590 |
| Oncology/Haematology  Chemotherapy  Anaemia  Malignant neoplasm of skin  Other oncology/haematology diagnosis | 423  194  41  27  161 |
| Gastroenterology  Alcoholic/Other Gastritis  Crohn’s Disease  Gastrointestinal Haemorrhage  Other gastroenterological diagnoses | 358  93  72  53  140 |
| Endocrinology  Diabetes (Type 1 & 2) with poor control  Type 2 Diabetes with foot ulcer  Type 2 Diabetes with ketoacidosis  Other endocrinology diagnoses | 166  61  38  9  58 |
| Rheumatology  Rheumatoid Arthritis  Gout  Systemic Lupus Erythematosus  Other rheumatological diagnoses | 182  61  49  17  55 |
| Surgery (including urology/vascular)  Non-specific abdominal pain  Cholecystitis  Pancreatitis  Other surgical diagnoses | 864  97  96  96  575 |
| Obstetrics and Gynaecology  Delivery  Management of diabetes arising during pregnancy  False Labour  Other obstetric and gynaecological diagnoses | 1653  376  111  110  1056 |
| Orthopaedics  Fractures  Lower back pain  Complication of joint prosthesis  Other orthopaedic diagnoses | 465  174  26  22  243 |
| Dental  Dental caries  Periapical abscess without sinus  Other dental diagnoses | 50  30  10  10 |
| Dermatology  Skin malignancy/melanocytic naevi  Bullous disorders  Urticaria  Other dermatological diagnoses | 23  4  3  3  13 |
| Ophthalmology  Cataracts  Injury/Contusion to the eye  Ophthalmic complication of Type 2 Diabetes  Other ophthalmological diagnoses | 70  31  19  6  14 |
| Paediatric  Pre-term/Low birth weight disorder  Syndrome of infant of a mother with Diabetes  Respiratory disorders  Other paediatric diagnoses | 59  19  13  5  22 |
| Psychiatry  Psychotic disorder/Schizophrenia  Depressive disorder/Suicidal ideation  Acute stress disorder/Anxiety  Other psychiatric diagnoses | 199  48  42  22  87 |
| Miscellaneous diagnoses  Non-specific chest pain  Alcohol intoxication and associated mental/behavioural disorder  Ascites  Fluid overload  Iron deficiency anaemia  Pain (musculoskeletal-related)  Rehabilitation  Electrolyte disturbance  Medications adverse reactions  Syncope/collapse  Fever  Other miscellaneous diagnoses | 1346  334  151  75  58  58  53  41  36  34  32  30  444 |
